# Effect of Annealing Temperature on the Optical Spectra of CdS Thin Films Deposited at Low Solution Concentrations by Chemical Bath Deposition (CBD) Technique

**DOI:** 10.3390/ijms12021293

**Published:** 2011-02-22

**Authors:** Zahid Rizwan, Azmi Zakaria, Mohd Sabri Mohd Ghazali, Atefeh Jafari, Fasih Ud Din, Reza Zamiri

**Affiliations:** 1 Department of Physics, Faculty of Science, Universiti Putra Malaysia, 43400 UPM Serdang, Selangor, Malaysia; E-Mails: zahidrizwan64@gmail.com (Z.R.); mgm.sabri@gmail.com (M.S.M.G.); atefeh.j87@gmail.com (A.J.); uddin.fasih@gmail.com (F.U.D.); zamiri.r@gmail.com (R.Z.); 2 Advanced Materials and Nanotechnology Laboratory, Institute of Advanced Technology, Universiti Putra Malaysia, 43400 UPM Serdang, Selangor, Malaysia

**Keywords:** chemical bath deposition, cadmium sulphide, window layer, air-annealing

## Abstract

Two different concentrations of CdCl_2_ and (NH_2_)_2_CS were used to prepare CdS thin films, to be deposited on glass substrate by chemical bath deposition (CBD) technique. CdCl_2_ (0.000312 M and 0.000625 M) was employed as a source of Cd^2+^ while (NH_2_)_2_CS (0.00125 M and 0.000625 M) for S^2−^ at a constant bath temperature of 70 °C. Adhesion of the deposited films was found to be very good for all the solution concentrations of both reagents. The films were air-annealed at a temperature between 200 °C to 360 °C for one hour. The minimum thickness was observed to be 33.6 nm for film annealed at 320 °C. XRD analyses reveal that the films were cubic along with peaks of hexagonal phase for all film samples. The crystallite size of the films decreased from 41.4 nm to 7.4 nm with the increase of annealing temperature for the CdCl_2_ (0.000312 M). Optical energy band gap (*E_g_*), Urbach energy (*E_u_*) and absorption coefficient (*α*) have been calculated from the transmission spectral data. These parameters have been discussed as a function of annealing temperature and solution concentration. The best transmission (about 97%) was obtained for the air-annealed films at higher temperature at CdCl_2_ (0.000312 M).

## 1. Introduction

Many techniques, such as electro-deposition, vacuum evaporation, sputtering, radio frequency, pulsed laser evaporation, molecular beam epitaxy (MBE), metal organic chemical vapor deposition (MOCVD), spray pyrolysis deposition (SPD), close-spaced sublimation (CSS), successive ionic layer adsorption and reaction (SILAR), Micelle method and chemical bath deposition (CBD), have been used to develop thin films for a long time. These techniques have also been used for the preparation of CdS films. Among these techniques, CBD has become an attractive route due to its simplicity, being inexpensive and having large surface area deposition at low temperature. This technique is also reported to offer an excellent control to deposit uniform thin films, and is also known as the solution growth technique or chemical deposition technique. CBD has been used to grow CdS films since the 1960s [1–3]. This technique also enhances the performance of CdS window layer as compared to other film growing techniques. Moreover, these films are composed of closely packed nanocrystals (NCs) which make them attractive for basic and applied research of NCs [4]. The highest efficiency was obtained with the use of the CBD technique to deposit thin films of CdS as a window layer, however preparing the film at very low concentration has not yet been discussed before. High efficiency was also obtained when the CBD technique was used to grow the buffer layer for CdTe and CIGS solar cells [5,6]. It is also used in the fabrication of other electronic and optoelectronic devices [7,8]. CdS is an excellent heterojunction partner for p-type CdTe, CuInSe_2_, Cu(In,Ga)Se_2_ (CIGS) because of the wide optical band gap (2.42 eV). CdS is also an important material due to its novel properties like photoconductivity, high index of refraction (2.5) and its high electron affinity [9,10]. The substrate is immersed in a bath of alkaline aqueous solution of chemicals. The ions Cd^2+^ and S^2−^ resulting from the chemical reaction in the bath solution grow CdS thin films on the immersed substrate [11,12]. Control of temperature, film deposition time, concentrations of the reactive which provide Cd^2+^ and S^2−^ ions for chemical reaction, pH of aqueous solution is required for the deposition of CdS film on the immersed substrate in the chemical bath deposition technique.

CdS film develops through a series of chemical reactions [13]. Firstly, cadmium salt hydrolyses to provide free Cd^2+^ ions. These cadmium ions react with ammonia molecules supplied by the ammonium salt to form the cadmium *n*-amine complex where *n* = 1, 2, 3…6.

$${\text{Cd}}^{2+}+n{\text{NH}}_{3}⇌{\text{Cd}{\left({\text{NH}}_{3}\right)}_{n}}^{2+}$$

Secondly, hydroxyl radical (OH)^1−^ promotes the thiourea hydrolysis to release the sulfur ions via successive chemical reactions,

$${\text{H}}_{2}\text{S}+2{\text{OH}}^{-}⇌{\text{S}}^{2-}+2{\text{H}}_{2}\text{O}$$

$${\left({\text{NH}}_{2}\right)}_{2}\text{CS}\to {\text{H}}_{2}\text{S}+{\text{CN}}_{2}{\text{H}}_{2}$$

$${\text{Cd}}^{2+}+{\text{S}}^{2-}\to \text{CdS}$$

Finally, the presence of the free ions of Cd^2+^ and S^2−^ enables CdS to be made.

In this study, the CBD technique has been used to deposit nanostructured CdS thin films on microscopic glass slides at very low solution concentrations of CdCl_2_ (0.000312 M) with thiourea (0.000625 M), and secondly CdCl_2_ (0.000625 M) with thiourea (0.000125 M). The optical and structural properties of CdS films are discussed as a function of annealing temperature and solution concentration.

## 2. Experimental

All the reagents and solvents used were of analytical grade and procured from Alfa Aesar (Malaysia). Solvents were of 99.9% purity and were used without further purification. Glasswares (Pyrex, Germany) used were soaked overnight in 20% nitric acid solution, followed by rinsing with double distilled deionized water and oven drying at 70 °C for 1 h.

Cadmium chloride and thiourea were used to grow CdS films on microscopic glass slides (76 × 25 × 1.2 mm) as substrate. The substrates were cleaned in acetone and ethanol ultrasonically for 20 min. Then substrates were washed with double deionized water and dried under N_2_ atmosphere. Solutions of two different concentrations, concentration A = CdCl_2_ (0.000625 M), thiourea (0.00125 M) and concentration B = CdCl_2_ (0.000312 M), thiourea (0.000625 M), were prepared in doubly distilled deionized water by continuous stirring at room temperature to deposit CdS films.

Six samples of each solution concentration (A and B) were prepared by mixing solutions of CdCl_2_ and (NH_2_)_2_CS. These solutions were placed separately in a water bath using a digital hot plate and the temperature was raised up to 65 °C while stirring. Ammonia (NH_3_) in aqueous solution was used as a complexing agent. It was added drop by drop in CdCl_2_ solution to dissolve the white precipitate of Cd(OH)_2_ under constant stirring conditions until the pH of the solution was adjusted to 11. Thiourea solution was added in CdCl_2_ solution in 30 s under vigorous stirring. The temperature of the resulting clear solution was further raised to 70 °C followed by immersing cleaned substrates vertically in the solution using special Teflon holders. The container was covered to avoid the evaporation of ammonia. Deposited substrates were washed in deionized water ultrasonically to remove the loosely adhered CdS particles and were dried at ambient conditions. Deposited film samples were divided into six sets. One sample from each solution concentration (A and B) was characterized as-deposited (A_0_ and B_0_). The other samples were air annealed in the temperature range (200–360 °C) for 1 h at a heating and cooling rate of 4 °C min^−1^. The samples ware characterized as for annealing temperature 200 °C (A_200_ and B_200_), 240 °C (A_240_ and B_240_), 280 °C (A_280_ and B_280_), 320 °C (A_320_ and B_320_) and 360 °C (A_360_ and B_360_).

Thickness of the films was measured by an Ellipsometer (DRE-Dr. RISS ELX-02C). Cu K_α_ radiation (λ = 1.540598 Å) with PANalytical (Philips) X’Pert Pro PW1830 was used for XRD analysis. The XRD data were analyzed by X’Pert High Score software for the identification of the crystalline phases in the films. Crystallite size (*D*) was determined using Scherer formula,

(1) $$D=\frac{K(\lambda )}{\beta \;\text{cos}\;\theta }$$

where β is the full width at half maximum (FWHM in radians) of the X-ray diffracted peak corrected for instrumental broadening and θ is Bragg angle, λ is the wavelength of X-ray, K is Scherer constant; taken as 0.94 for the calculations [14,15].

The optical transmission (*T*) data was measured by double beam spectrophotometer (Shimadzu) over the wavelength range of 350 to 1100 nm. The absorption coefficient (*α*) was calculated using the Equation,

(2) $$\alpha =\frac{\text{ln}(\frac{1}{T})}{d}$$

Optical energy band gap (*E_g_*) can be calculated using absorption coefficient (*α*),

(3) $$\alpha =\frac{A{\left(h\upsilon -E_{g}\right)}^{n}}{h\upsilon }$$

where *A* is constant, *hυ* is photon energy, *n* is 0.5 for direct band gap materials as CdS is a direct band gap material [16,17]. (*αhυ*)^2^ is plotted as a function of *hυ*. The linear portion of the curve extrapolated to (*αhυ*)^2^ = 0, gives the value of *E*_g_. The absorption coefficient (*α*) shows a tail for sub-band gap energy. The Urbach energy (*E_u_*), associated with the width of the tail was measured from the formula [17],

(4) $$\alpha =\alpha_{o}e^{h\upsilon /E_{u}}$$

where *α*_o_ is constant. The inverse of the slope from the plot of ln*α* *versus hυ* gives the value of Urbach energy (*E_u_*).

## 3. Results and Discussion

**Figure 1.**
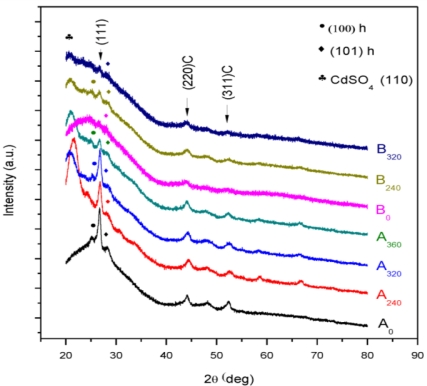
XRD pattern of CdS film for different annealing temperatures.

CdS films prepared from a reaction mixture containing cadmium chloride and thiourea for both molar concentrations (A and B) show polycrystalline nature [18,19] as shown in the XRD pattern, Figure 1. The XRD graphs are scaled to a small size and seven graphs are presented in one figure, many of the small peaks are lost in this process. Here, all of the peaks that were observed are described in detail in Table 1. Both samples without any heat treatment are polycrystalline and a mixture of cubic and hexagonal phase of CdS [18–20].

**Figure 2.**
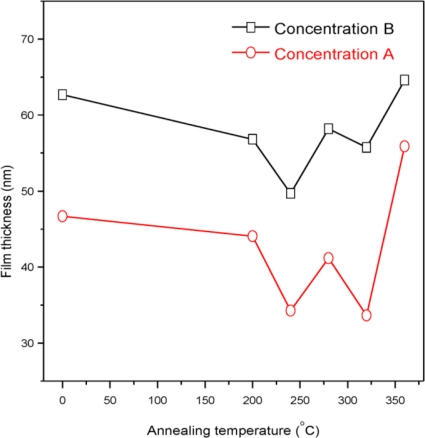
Variation of film thickness with different annealing temperatures.

In all samples except A_0_ and B_0_, two peaks were observed at 2θ = 20.8511° and 21.5199° (ref: 00-036-0898) belonging to (110), (020) orthorhombic CdSO_4_. It is also observed that the preferred orientation is (111) which is (111) due to the controlled nucleation process occurring in the growing film. This suggests the slow growth rate of the film deposition [21]. Thickness of the film sample B_0_ is 62.7 nm and is further decreased with the increase of the annealing temperature (Figure 2). The thickness is about 64.6 nm for the sample B_360_. It was observed that the thickness of the samples prepared from the solution concentration A is lower than that of the samples prepared from solution concentration B for all annealing temperatures. The minimum thickness 33.6 nm was observed for the sample A_320_. This indicates the thickness of the film is lower when the higher solution concentration is used.

**Figure 3.**
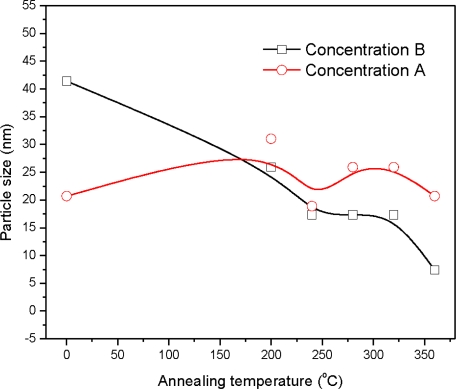
Variation of crystallite size with the annealing temperature.

The average crystallite size of the film sample A_0_ is 20.7 nm. The crystallite size of the sample A_200_ is 31 nm and is further reduced to 20 nm for the sample A_360_, Figure 3. The average crystallite size of the film sample B_0_ is 41.4 nm and is reduced to 7.4 nm for the sample B_360_. This indicates a decrease in crystallite size with the increase of annealing temperature, suggesting an increase in crystallinity with annealing temperature. This situation was not observed for the samples prepared from the solution concentration A, which is higher than that of solution concentration B. This indicates the films developed at higher concentration were more crystalline in nature before annealing.

**Figure 4.**
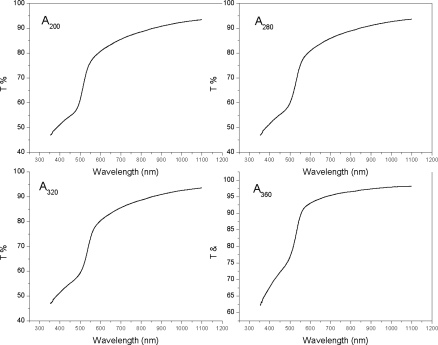
Transmittance spectra for different annealing temperatures for concentration A.

**Figure 5.**
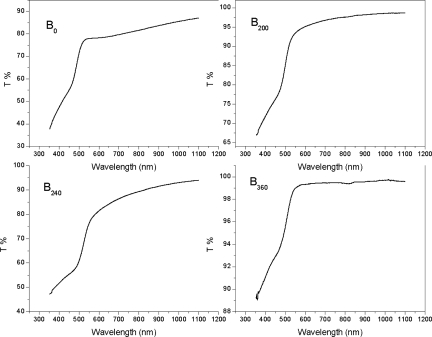
Transmittance spectra for different annealing temperatures for concentration B.

The transmittance spectra of CdS films were recorded over 350 to 1100 nm (Figures 4 and 5). The spectra showed transmittance dependence of film on the wavelength at a different annealing temperature. The transmittance (T%) is about 89% at wavelength of 550 nm for the sample A_360_ and is increased to 97% at the wavelength 850 nm, Figure 4. It is slightly increased with the further increase of wavelength. The transmittance (T%) is about 98% at wavelength of 550 nm for the sample B_360_ and remains approximately constant with the further increase of the wavelength up to 1100 nm, Figure 5.

**Figure 6.**
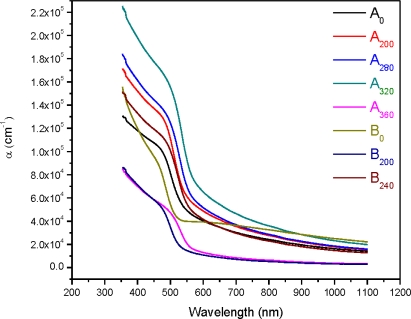
Absorption coefficient (*α*) for different annealing temperatures.

It was observed that transmission spectra shift towards the lower wavelength range for the sample B_360_, which indicates the increase in the optical energy band gap. This suggests the higher concentration of the solution (concentration A) has better crystallinity as compared to the samples that are prepared from the lower concentration (concentration B) at the annealing temperature 360 °C. The transmittance is about 80% at wavelength of 550 nm for the sample A_0_ and is increased to 91% at the wavelength 850 nm. It is slightly increased with the further increase of wavelength. The transmittance is 78% at wavelength of 550 nm for the sample B_0_ and is slightly increased with the further increase of wavelength up to 1100 nm. The transmission spectrum is observed to shift towards the lower wavelength range for the sample B_0_. This shift of spectra indicates the increase in the optical energy band gap (*E_g_*), as shown in Figure 8 [22]. This suggests the higher concentration of the solutions (concentration A) has the better crystallinity as compared to the samples which are prepared from the lower concentration solutions (concentration B) for the as-deposited CdS thin films. The variation of the optical absorption coefficient (*α*) with wavelength for different annealing temperatures and solution concentrations is shown in Figure 6.

**Figure 7.**
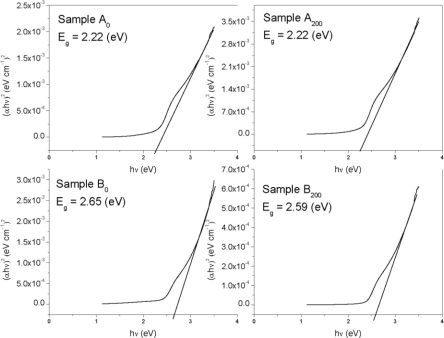
Variation of the optical energy band gap (*E_g_*) with photon energy (*hυ*) at different concentrations and annealing temperatures.

**Figure 8.**
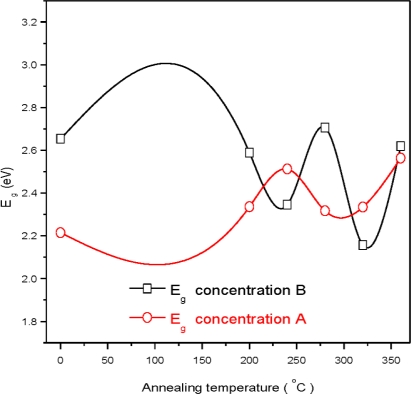
Variation of the optical energy band gap (*E_g_*) at different annealing temperatures.

**Figure 9.**
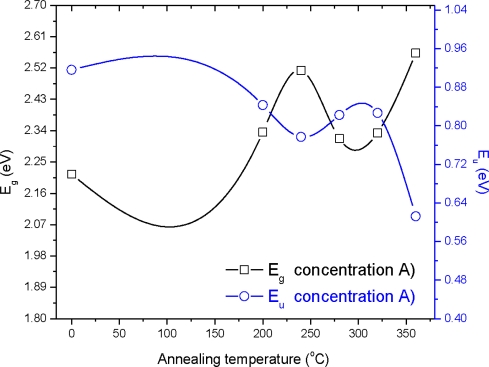
Variation of the optical energy band gap (*E_g_*) and Urbach energy (*E_u_*) with annealing temperature for concentration A.

**Figure 10.**
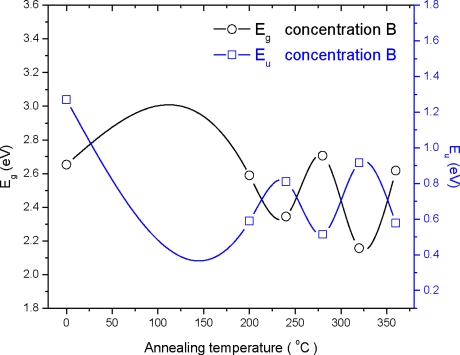
Variation of the optical energy band gap (*E_g_*) and Urbach energy (*E_u_*) with annealing temperature for concentration B.

**Figure 11.**
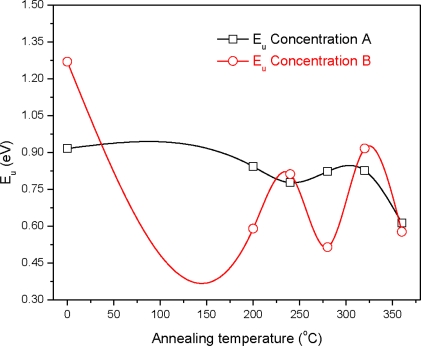
Variation of Urbach energy (*E_u_*) with annealing temperature for concentration A and B.

The graph (*αhυ*)^2^ *versus* photon energy (*hυ*) is shown in Figure 7. The value of the optical energy band gap (*E_g_*) is 2.22 eV for sample A_0_. The value of the optical energy band gap (*E_g_*) is 2.65 eV for sample B_0_ Figure 8. It was observed that there is a prominent change in the value of *E_g_* for the samples A_0_ and B_0_. This suggests that the crystallinity of the films increased with the increase of the solution concentration. It is clear from Figure 2 that the film thickness is also increased with the decrease of solution concentration. Particle size also increased with the decrease of solution concentration. It is supposed that loosely adherent collides are formed with the decrease of the concentration. This may be the reason why the value of *E_g_* is increased. It is also observed that the value of *E_g_* for solution concentration A (higher concentration) increases with the increase of annealing temperature indicating that the crystallinity is enhanced with annealing temperature. The trend in the value of *E_g_* decreases for the samples prepared from solution concentration B (lower concentration). This suggests a decrease in the crystallinity for very low concentrations. *E_u_* is Urbach energy and is shown in Figures 9 and 10. *E_u_* is also known as band tail width and is due to the disorder in the thin film material. Its variation with annealing temperature is shown in Figure 11 for concentration A and B. The variation of bond length and bond angle from their standard value in the crystalline material is called disorder [23,24]. It is clear that the optical band gap is opposite to the disorder. This behavior indicates that the obtained optical band gap is governed with the disorder variation in the CdS films.

## 4. Conclusions

The CdS films were deposited by the CBD technique for the different solution concentrations at constant bath temperature. XRD analysis shows that the films were in cubic phase along with the hexagonal CdS at both solution concentrations. The crystallite size varied with the annealing temperature. Optical transmission 75–97% was observed at the wavelength 850 nm. The optical transmittance varied with the annealing temperature. The optical band gap was governed with the disordering phenomena in CdS films.

## Figures and Tables

**Table 1.** A summary of XRD data for all samples.

**Sample**	**2θ (Deg.) (hkl)**	**CdS Structure**
A_0_	26.7277°(111), 44.1346°(220), 52.3619°(311)	cubic (ref: 01-080-0019)
	25.1618°(100), 28.2306°(101), 48.1229°(103)	hexagonal (ref: 01-080-0006)
B_0_	26.4962°(111), 43.9355°(220), 51.9702°(311)	cubic (ref: 01-075-1546)
	28.1727°(101)	hexagonal (ref: 00-065-3414).
A_200_	26.8079°(111), 44.4133°(220), 52.7364°(311)	cubic (ref: 01-075-0581)
	28.6450°(101), 58.4937°(202), 66.7578°(203)	hexagonal (ref: 01-080-0006).
B_200_	26.7070°(111), 44.2175°(220), 52.4116°(311)	cubic (ref: 01-080-0019)
	24.6784°(100), 28.3841°(101), 58.4548°(202), 66.6981°(203)	hexagonal (ref: 01-089-2944).
A_240_	26.8176°(111), 30.8886°(220), 44.4089°(311	cubic (ref: 01-075-1546)
	58.5947°(202), 66.8296°(203)	hexagonal (ref: 01-080-0006).
B_240_	26.6935°(111), 44.0913°(220), 52.3710°(311)	cubic (ref: 01-075-1546)
	25.0745°(100), 28.4921°(101)	hexagonal (ref: 00-001-0783).
A_280_	26.7905°(111), 30.7475°(200), 44.4319°(220), 52.5711° (311), 73.2311°(420)	cubic (ref: 01-080-0019)
	24.7269°(100), 28.3734°(101), 48.0309°(103), 58.6591° (202), 66.8441°(203)	hexagonal (ref: 01-077-2306).
B_280_	25.5707°(111), 30.3946°(200), 44.1649°(220)	Cubic (ref: 00-042-1411)
	28.1785°(101), 47.5271°(103), 58.3791°(202), 66.5891°(203)	hexagonal (ref: 01-080-0006).
A_320_	26.8012°(111), 44.2445°(220), 52.5047°(311)	cubic (ref: 01-080-0019)
	25.3195°(100), 28.5660°(101), 58.3476°(202)	hexagonal (ref: 00-001-0783).
B_320_	26.5834°(111), 44.0583°(220), 52.1528°(311)	cubic (ref: 01-080-0019)
	28.4385°(101)	hexagonal (ref: 01-075-1545).
A_360_	26.7119°(111), 43.9950°(220), 52.2836°(311	cubic (ref: 01-080-0019)
	24.9576 °(100), 28.2761°(101), 58.2849°(202)	hexagonal (ref: 01-080-0006).
B_360_	43.7486°(220)	cubic (ref: 00-065-2887).
	24.6729°(100)	hexagonal (ref: 00-006-0314)
